# Prevalence of the second mesiobuccal canal of maxillary molars in the South American population: a systematic review and meta-analysis of CBCT studies

**DOI:** 10.3389/fdmed.2026.1928424

**Published:** 2026-08-31

**Authors:** Kevin José Poclin-Quispe, Heber Isac Arbildo-Vega, Fray L. Becerra-Suarez, Gabriel Guerra-Gonzales, David Yeret Rodríguez-Salazar, Franz Tito Coronel-Zubiate

**Affiliations:** 1 Faculty of Health Sciences, School of Dentistry, Universidad Privada Norbert Wiener, Lima, Peru; 2 Grupo de Investigación en Inteligencia Artificial (UMA-AI), Facultad de Ingeniería, Universidad Privada Norbert Wiener, Lima, Peru; 3 Faculty of Human Medicine, Human Medicine School, Universidad San Martín de Porres, Chiclayo, Peru; 4 Faculty of Post Graduate, Universidad Nacional Toribio Rodríguez de Mendoza de Amazonas, Chachapoyas, Peru; 5 Grupo de Investigación en Inteligencia Artificial (UMA-AI), Facultad de Ingeniería y Negocios, Universidad Privada Norbert Wiener, Lima, Peru; 6 Graduate School, Universidad Señor de Sipán, Chiclayo, Peru; 7 Faculty of Health Sciences, Universidad Nacional Toribio Rodríguez de Mendoza de Amazonas, Chachapoyas, Peru

**Keywords:** CBCT, endodontics, MB2, mesiobuccal canal, prevalence, review, South America

## Abstract

**Background:**

The objective of this systematic review and meta-analysis was to determine the prevalence of the second mesiobuccal (MB2) canal in permanent maxillary molars within the South American population, evaluated exclusively through cone-beam computed tomography (CBCT).

**Methods:**

Following the PRISMA guidelines, an electronic search was conducted across PubMed, Scopus, Web of Science, Embase, SciELO, LILACS, and gray literature until May 2026. Observational studies published from 1990 onwards were included. A meta-analysis of proportions was executed using a random-effects model. The risk of bias was assessed with the JBI tool, and the certainty of evidence using the GRADE framework.

**Results:**

A total of 23 observational studies were included. The pooled overall prevalence of the MB2 canal was 59% (95% CI: 53%–66%). In the tooth-specific analysis, prevalence reached 66% in first molars and 41% in second molars. Significant sexual dimorphism was evident in second molars, where males presented a 35% higher probability of possessing the canal (OR = 1.35). The GRADE analysis indicated a High certainty for the overall and first molar prevalences, and Moderate for second molars.

**Conclusions:**

The MB2 canal is a highly prevalent anatomical variation in South America, constituting the anatomical norm in first molars. The clinical use of CBCT is crucial for its diagnostic prediction and successful management.

**Systematic Review Registration:**

DOI: 10.17605/OSF.IO/KRG4Z.

## 1 Introduction

The long-term success of endodontic treatment depends on accurate knowledge of root canal anatomy, thorough canal identification, effective chemomechanical disinfection, and adequate obturation (1, 2). Human maxillary molars present one of the most complex root canal anatomies, frequently exhibiting a second canal in the mesiobuccal root (MB2), whose missed detection has been recognized as an important cause of persistent infection and post-treatment disease (3–5).

The MB2 canal is often difficult to locate because its orifice may be hidden beneath dentin projections or located within narrow developmental grooves (5). Conventional periapical radiography frequently underestimates its prevalence because of anatomical superimposition (6). Consequently, clinicians should be aware of its frequent occurrence and apply appropriate diagnostic strategies to improve canal detection and treatment outcomes.

Advances in imaging technologies have improved the *in vivo* detection of the MB2 canal. Cone-beam computed tomography (CBCT) provides three-dimensional visualization without geometric distortion and has become the preferred non-invasive imaging modality for evaluating root canal anatomy (5, 7, 8). During treatment, the use of an operating microscope combined with ultrasonic tips further improves canal localization, although calcifications and anatomical complexity may still limit complete canal negotiation (1, 9–11).

Previous studies have reported substantial variability in MB2 prevalence, ranging from approximately 48% to more than 90% among different populations (12). Global meta-analyses estimate pooled prevalences of 60%–69.6% in maxillary first molars and 23.3%–39.0% in second molars (4.5). This variability has been attributed to genetic, anthropological, and methodological factors, highlighting the importance of evaluating population-specific prevalence.

Despite the available literature, the factors influencing MB2 channel prevalence remain incompletely understood. Previous studies have reported variations according to ethnicity and geographic origin, suggesting that population characteristics may influence root canal morphology (5). Differences according to sex and age have also been investigated, although findings remain inconsistent across studies (4, 5, 12, 13). Furthermore, methodological differences, including CBCT acquisition parameters, image resolution, and sample characteristics, may contribute to the wide variability in reported prevalence estimates. These factors highlight the need for population-specific evidence to better characterize the anatomical distribution of the MB2 canal.

South America represents a particularly relevant region because its population exhibits considerable genetic admixture that may influence root canal morphology (14). Although several CBCT studies have been conducted in Brazil, Chile, Peru, Venezuela, and other countries, their reported prevalences vary substantially, and no quantitative synthesis has specifically summarized the available evidence for this region (12, 15–19).

Therefore, this systematic review and meta-analysis aimed to determine the prevalence of the MB2 canal in permanent maxillary molars in South American populations using CBCT. The findings are expected to provide an updated regional estimate to support clinical decision-making and improve the understanding of anatomical variability relevant to contemporary endodontic practice.

## 2 Article types

Systematic review.

## 3 Materials and methods

### 3.1 Protocol and registration

This systematic review was conducted in accordance with the Preferred Reporting Items for Systematic Reviews and Meta-Analyses Protocols (PRISMA-P) guidelines (20). The review protocol was prospectively registered in the Open Science Framework (OSF) under the DOI 10.17605/OSF.IO/KRG4Z, which is publicly accessible. Given the observational nature of this systematic review, institutional ethical approval was not required.

No publication year restrictions were applied to maximize the sensitivity of the search strategy. Although CBCT became clinically available after the late 1990s, no studies published before this period met the eligibility criteria, and all included studies were published after the introduction of this imaging modality.

### 3.2 Focused question and PICO framework

The central research question was structured using the CoCoPop framework (Condition, Context, and Population), specifically adapted for prevalence reviews, as detailed below:

- Condition: The prevalence or absolute frequency of the MB2.
- Context: The South American population (specifically encompassing Argentina, Bolivia, Brazil, Chile, Colombia, Ecuador, Guyana, Paraguay, Peru, Suriname, Uruguay, and Venezuela) evaluated exclusively through CBCT imaging.
- Population: Fully developed permanent maxillary first and second molars in human patients.

**Focused question:** What is the estimated prevalence of the MB2 in permanent maxillary first and second molars within the South American population, as assessed by CBCT?

### 3.3 Information sources and search strategy

A comprehensive electronic literature search was conducted across six primary databases: PubMed, Scopus, Web of Science, Embase, SciELO, and LILACS. To mitigate publication bias, gray literature was also systematically explored using Google Scholar and ProQuest Dissertations & Theses Global. Furthermore, a manual screening of the reference lists of all included studies was performed to identify any relevant supplementary publications. The search encompassed all available literature published up to May 2026.

All retrieved records were imported into the reference management software Zotero® (Center for History and New Media, Virginia, USA), where duplicate entries were systematically removed. The detailed search algorithms and strategies tailored to each respective database are outlined in Table 1.

**Table 1:** Search strategies for each database.

Database	Search strategy	Number of study
Pubmed	(((“Molar"[Mesh] OR “Maxilla"[Mesh]) OR (“Maxillary molar*” OR “Upper molar*” OR “First molar*” OR “Second molar*”)) AND ((“Dental Pulp Cavity"[Mesh] OR “Root Canal Therapy"[Mesh:NoExp]) OR (“Second mesiobuccal” OR “MB2” OR “MB-2” OR “mesio-buccal-2” OR “extra canal*” OR “root canal morphology” OR “canal configuration”)) AND ((“Cone-Beam Computed Tomography"[Mesh] OR “CBCT” OR “Cone beam”)) AND ((“South America"[Mesh] OR “Argentina” OR “Bolivia” OR “Brazil” OR “Chile” OR “Colombia” OR “Ecuador” OR “Guyana” OR “Paraguay” OR “Peru” OR “Suriname” OR “Uruguay” OR “Venezuela” OR “South America*” OR “Latin America*”)))	129
Scopus	(TITLE-ABS-KEY ((“Maxillary molar*” OR “Upper molar*” OR “First molar*” OR “Second molar*”)) AND TITLE-ABS-KEY ((“Second mesiobuccal” OR “MB2” OR “MB-2” OR “extra canal*” OR “root canal morphology”)) AND TITLE-ABS-KEY ((“CBCT” OR “Cone beam”)) AND TITLE-ABS-KEY ((“South America” OR “Argentina” OR “Bolivia” OR “Brazil” OR “Chile” OR “Colombia” OR “Ecuador” OR “Guyana” OR “Paraguay” OR “Peru” OR “Suriname” OR “Uruguay” OR “Venezuela” OR “Latin America”)))	12
Web of Science	((“maxillary molar*” OR “upper molar*” OR “maxillary first molar*” OR “maxillary second molar*” OR “upper first molar*” OR “upper second molar*”) AND (“Second mesiobuccal” OR “mesiobuccal canal*” OR MB2 OR “MB 2” OR “MB-2” OR “extra canal*” OR “additional canal*” OR “root canal morphology” OR “canal morphology”) AND (CBCT OR “Cone beam” OR “Cone-beam” OR “Cone beam computed tomography” OR “Cone-beam computed tomography”) AND (“South America” OR “Latin America” OR Argentina OR Bolivia OR Brazil OR Brasil OR Chile OR Colombia OR Ecuador OR Guyana OR Paraguay OR Peru OR Perú OR Suriname OR Uruguay OR Venezuela))	39
Embase	(‘molar'/exp OR ‘maxillary molar*’ OR ‘upper molar*’ OR ‘maxillary first molar*’ OR ‘maxillary second molar*’ OR ‘upper first molar*’ OR ‘upper second molar*’) AND (‘root canal anatomy'/exp OR ’second mesiobuccal canal*’ OR ‘mesiobuccal canal*’ OR mb2 OR ‘mb-2’ OR ‘mb 2’ OR ‘extra canal*’ OR ‘additional canal*’ OR ‘root canal morphology’ OR ‘canal morphology’) AND (‘cone beam computed tomography'/exp OR cbct OR ‘cone beam’ OR ‘cone-beam’ OR ‘cone beam computed tomography’ OR ‘cone-beam computed tomography’) AND (‘South America'/exp OR ‘Latin America'/exp OR Argentina OR Bolivia OR Brazil OR Brasil OR Chile OR Colombia OR Ecuador OR Guyana OR Paraguay OR Peru OR Perú OR Suriname OR Uruguay OR Venezuela)	62
SciELO	((ti:(”molar”)) OR (ab:(”molar”)) AND (ti:(”MB2” OR “mesiobuccal” OR “mesiovestibular”)) OR (ab:(”MB2” OR “mesiobuccal” OR “mesiovestibular”))) AND ((tw:(”CBCT” OR “South America” OR “Sudamérica” OR “América do Sul”)))	83
LILACS	(mh:(”Molar” OR “Maxila”) OR tw:(”Molar maxilar” OR “Molar superior” OR “Primeiro molar” OR “Segundo molar”)) AND (mh:(”Cavidade da Polpa Dentária” OR “Anatomia do Conduto Radicular”) OR tw:(”Segundo conduto mesiovestibular” OR “Segundo conducto mesiobuccal” OR “MB2” OR “MB-2” OR “Morfologia do canal”)) AND (mh:(”Tomografia Computadorizada de Feixe Cônico”) OR tw:(”CBCT” OR “Cone beam”)) AND (mh:(”América do Sul” OR “Argentina” OR “Bolívia” OR “Brasil” OR “Chile” OR “Colômbia” OR “Equador” OR “Guiana” OR “Paraguai” OR “Peru” OR “Suriname” OR “Uruguai” OR “Venezuela”))	24
Proquest Dissertations and Theses	TI,AB(”Maxillary molar*” OR “upper molar*”) AND TI,AB(”Second mesiobuccal” OR “MB2” OR “MB-2” OR “extra canal*”) AND NOFT(”CBCT” OR “Cone beam”) AND NOFT(”South America” OR “Argentina” OR “Brazil” OR “Peru” OR “Chile” OR “Colombia”)	3
Google Scholar	"maxillary molar” (“second mesiobuccal” OR MB2) CBCT (“South America” OR Argentina OR Brazil OR Chile OR Colombia OR Peru OR Ecuador)	260

### 3.4 Data management and selection process

The study screening and selection processes were managed utilizing the Rayyan® web application (Qatar Computing Research Institute, Qatar). Study selection was executed in two systematic phases. During the primary phase, two independent reviewers (F.C.-Z. and F.C.-O.) screened the titles and abstracts of all retrieved records. In the secondary phase, the full texts of potentially eligible studies were independently evaluated by the same two reviewers. Any discrepancies at either stage were resolved through consensus discussion or, if necessary, consultation with a third reviewer (J.M.).

To be considered eligible for inclusion, studies were required to strictly meet the following criteria:

- Study Design: Observational studies (cross-sectional, descriptive, retrospective, or prospective) reporting prevalence data.
- Population: Male and female patients, without ethnic restrictions, residing in South American countries (Argentina, Bolivia, Brazil, Chile, Colombia, Ecuador, Guyana, Paraguay, Peru, Suriname, Uruguay, and Venezuela) presenting with fully developed permanent maxillary first and second molars.
- Intervention/Examination: Studies in which the root canal system was evaluated exclusively via CBCT.
- Outcomes: Studies reporting the absolute frequency or percentage of the MB2 canal's presence, preferably stratified by molar type (first or second) and anatomical classification (e.g., Vertucci or Ahmed).
- Tooth Status: Teeth demonstrating complete root formation with closed apices.
- Language: No publication language restrictions were applied.
- Timeframe: Studies published from the year 1990 onwards.

Studies were explicitly excluded if they met any of the following criteria:

- Geography: Populations residing outside of South America or South American emigrants in other continents, to eliminate potential environmental, dietary, or external genetic admixture biases.
- Alternative Methodology: Imaging or diagnostic methodologies other than clinical CBCT (e.g., sole reliance on periapical radiography, clearing techniques, micro-CT unless analyzed separately, or clinical microscopic inspection lacking tomographic support).
- Previous Endodontic Treatment: Molars with prior root canal therapy, as obturation materials generate radiographic artifacts that obscure the native anatomy.
- Anomalies or Pathologies: Teeth exhibiting root resorption, extensive pulp chamber calcification, dens invaginatus, or root fractures.
- Specific Populations: Deciduous dentition or third molars.
- Image Quality: Studies employing a CBCT voxel size exceeding 0.3 mm, as this resolution is inadequate for the reliable identification of small accessory canals.
- Publication Type: Literature reviews, case reports, letters to the editor, *in vitro* studies (where CBCT was not performed under standard clinical conditions), expert opinions, systematic reviews, clinical trials, quasi-experimental designs, or animal studies.

### 3.5 Data collection process

Data extraction was performed independently and in duplicate by two reviewers (R.M. and C.Z.) utilizing a standardized, pre-piloted data collection form. The extracted information was subsequently cross-checked for consistency, and any disagreements were adjudicated by a third author (C.P.). The following variables were systematically recorded:

- Study Characteristics: Author(s), year of publication, country of origin, study period, geographic scope of data capture, and study design.
- Demographic Data: Total number of subjects (stratified by sex) and mean age (including range).
- Methodological Parameters: CBCT device model, voxel size (µm), and field of view (FOV).
- Anatomical Outcomes: Specific teeth evaluated, total number of teeth (stratified by sex), and the final prevalence of the MB2 canal (stratified by sex).

### 3.6 Risk of bias (RoB) assessment

The risk of bias (RoB) assessment was independently conducted by two calibrated reviewers (T.G. and H.V.), achieving near-perfect inter-rater reliability (k = 0.98). Any minor discrepancies were resolved through mediation by a third reviewer (H.A.).

The risk of bias of the included studies was independently assessed using the Joanna Briggs Institute (JBI) Critical Appraisal Checklist for Prevalence Studies (21). This tool consists of 9 domains that evaluate the sampling frame, sampling technique, sample size, description of subjects and setting, data analysis coverage, validity of diagnostic methods, standardization of measurement, statistical analysis, and response rate. Each question was rated as “Yes”, “No”, “Unclear”, or “Not applicable” (N/A).

For the overall risk of bias classification of each study, the percentage of affirmative responses (“Yes”) was calculated based solely on the applicable questions. Three bias categories were established:

- Low risk of bias: When the study achieved ≥70% of affirmative responses, indicating that it meets most methodological standards.
- Moderate risk of bias: When the study obtained between 50% and 69% of affirmative responses. In these cases, although some methodological details are missing, they do not invalidate the overall results.
- High risk of bias: When the study presented <50% of affirmative responses, reflecting serious methodological flaws.

### 3.7 Data synthesis

#### 3.7.1 Statistical model and prevalence estimation

The quantitative synthesis was conducted via a meta-analysis of proportions to establish the pooled prevalence of the MB2 canal in South America. Raw proportions were utilized rather than variance transformations (e.g., Freeman-Tukey arcsine transformation) because the data distribution was sufficiently distant from the extreme values of 0 and 1. Specifically, 87.5% of the included studies reported prevalences ranging from 30% to 80%, which ensured the mathematical stability of the estimates and facilitated a straightforward clinical interpretation.

Given the anticipated clinical and methodological heterogeneity, a random-effects model was employed. Between-study variance (*τ*^2^) was estimated using the restricted maximum likelihood (REML) method. Overall pooled estimates are presented alongside their respective 95% confidence intervals (CIs).

#### 3.7.2 Heterogeneity evaluation and subgroup analysis

Statistical heterogeneity was quantified using the I^2^ index, while its statistical significance was evaluated using Cochran's *Q* test. To systematically explore the sources of observed variability, the following analyses were conducted:

- Subgroup Analysis: Prevalence estimates were stratified by country, study design, field of view (FOV), sex, and risk of bias (low, moderate, or high).
- Meta-regression: A random-effects meta-regression was performed to assess the impact of temporal trends and voxel size on MB2 prevalence. Importantly, the chronological period of data collection (the exact year the study was executed) was utilized as a covariate rather than the publication year, allowing for a more accurate reflection of evolving clinical practices and technologies. For studies spanning multiple years, this temporal covariate was calculated as the midpoint between the study's start and end dates.

#### 3.7.3 Sensitivity analysis and cumulative meta-analysis

To evaluate the robustness of the pooled findings and determine the mathematical influence of individual studies, a sensitivity analysis was executed utilizing the “leave-one-out” method. Furthermore, a cumulative meta-analysis was performed—ordered chronologically by the study execution period—to monitor the stability of the pooled prevalence and the incremental gain in statistical precision over time.

#### 3.7.4 Publication bias

Potential publication bias was initially evaluated through the visual inspection of funnel plots for asymmetry. This asymmetry was subsequently quantified using Egger's and Harbord's regression tests, with a threshold of *p* < 0.05 considered indicative of significant small-study effects or publication bias.

#### 3.7.5 Statistical software

All statistical analyses were conducted utilizing STATA software, version 18.0 (StataCorp, College Station, TX, USA), specifically employing the built-in meta command suite for prevalence data synthesis and random-effects modeling.

### 3.8 Certainty of evidence (GRADE)

The overall certainty of the synthesized evidence was appraised following the Grading of Recommendations Assessment, Development and Evaluation (GRADE) framework. This assessment was structured and finalized utilizing the GRADEpro Guideline Development Tool (GDT) software (McMaster University and Evidence Prime Inc., Hamilton, ON, Canada).

## 4 Results

### 4.1 Review and selection of primary studies

**Figure 1:**
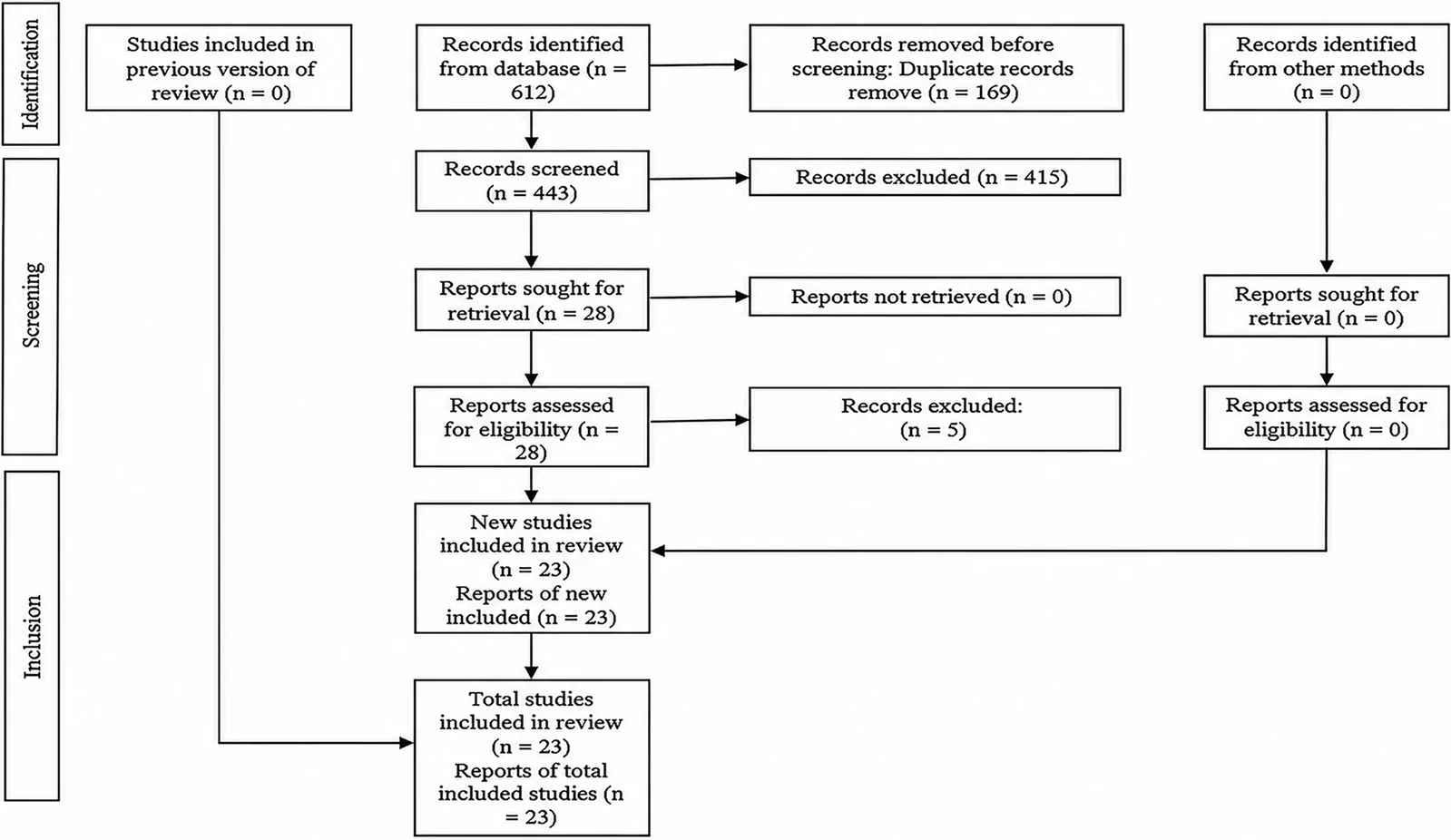
PRISMA flow diagram showing the selection process of studies included in the systematic review, from initial identification to final inclusion.

The comprehensive electronic database search initially yielded a total of 612 records. Following the systematic removal of duplicates, 443 unique references remained for evaluation. During the primary screening phase, an assessment of titles and abstracts identified 28 studies as potentially eligible, which were subsequently retrieved for full-text review. After a rigorous evaluation against the predefined inclusion and exclusion criteria, 5 articles were excluded, culminating in a final total of 23 studies included for qualitative and quantitative analysis. The specific justifications for these exclusions are detailed in Table 2, while the complete workflow of study identification, screening, eligibility assessment, and final inclusion is presented in the PRISMA 2020 flow diagram (Figure 1), in accordance with the PRISMA 2020 Statement.

**Table 2:** Reason for exclusion of studies.

Author	Reason for exclusion
Lilaj et al. (22)	No South America
Mirza et al. (23)	
Alnowailaty et al. (24)	
Zhang et al. (25)	
Ortiz Meneses et al. (26)	Inclusion of molars only with MB2

### 4.2 Review and characteristics of included studies

A total of 23 cross-sectional and retrospective observational studies (12, 14, 17, 18, 27–45), published between 2009 and 2025, were included in this systematic review. Regarding geographic distribution within South America, the majority of the research was conducted in Brazil (*n* = 13) and Chile (*n* = 7). Additional data were contributed by single studies from Argentina (*n* = 1) and Peru (*n* = 1), as well as one multi-center investigation that included distinct subpopulations from Brazil and Venezuela (Table 3).

**Table 3:** Characteristics of included studies.

Author(s)	Year	Country	Study period	Geographic scope of data capture	Design	Number of subjects (male/female)	Age average (range)	CBCT device	Voxel size (µm)	FOV	Classification of anatomical configuration	Teeth studied	Number of teeth (male/female)	Overall MB2 prevalence (male/female)
Blank-Gonçalves et al. (27)	2025	Brazil	2021–2024	Private radiology clinic	Cross-sectional and retrospective study	307 (184/123)	(≥18)	OP3D Pro CT (Instrumentarium, Kavo, Tuusula, Finlandia)	85	5 × 5 cm (Small)	Vertucci	Maxillary first molar	614 (368/246)	90 (42/58)
Borges et al. (28)	2025	Brazil	NR	Dental radiology diagnostic center, Parnaíba, Piauí	Cross-sectional	142 (50/92)	NR	Orthopantomograph OP300 3D, United State	250	6 × 4 cm (Small)	NR	Maxillary first molar	284 (100/184)	77.1 (55/47.8)
Rios et al. (29)	2023	Chile	2018	Private maxillofacial radiology center, Temuco	Cross-sectional	199 (71/128)	38 (12–72)	CS 8100 3D (Carestream, Atlanta, United State)	300	23 × 17 cm (Large)	Vertucci	Maxillary first molar	199 (71/128)	62.3
Dufey et al. (30)	2023	Chile	2014–2021	Dental clinic of the Andrés Bello University, Viña del Mar	Cross-sectional	262	(≥ 18)	I-CAT Vision Visualizer (Imaging Sciences International, Hatfield, United States)	500	NR	Vertucci	Maxillary first molar	524 (176/348)	63.7 (73.9/58.6)
												Maxillary second molar	524 (176/348)	20 (28.4/15.8)
Normando et al. (31)	2022	Brazil	2016–2019	Image clinic of the Christus University center, Fortaleza, Ceará	Cross-sectional and retrospective study	250 (125/125)	42 (10–69)	Eagle 3D (Dabi Atlante, Ribeirão Preto, São Paulo, Brazil)	NR	12 × 7.5 cm (Large)	NR	Maxillary first molar	382 (196/186)	76.4 (79.6/73.1)
												Maxillary second molar	405 (214/191)	41.7 (45.8/37.2)
Magalhães et al. (32)	2022	Brazil	2016–2017	Private dental radiology clinic, Federal University of Pernambuco, Recife, Pernambuco	Cross-sectional and retrospective study	414 (176/238)	47.5 (10–89)	i-CAT Next Generation CBCT scanner (Imaging Sciences International, Hatfield, Pennsylvania, United State)	300	17 × 13 cm (Large)	Weine and Vertucci	Maxillary first and second molar	1000 (438/562)	68.4 (71/66.4)
Benazzo et al. (33)	2020	Argentina	NR	Clinic of the Faculty of Dentistry of the University of Buenos Aires, Buenos Aires	Cross-sectional and retrospective study	148	30 ± 16 (9–72)	Planmeca Romexis CT Scanner, Planmeca Helsinki, Finland	NR	NR	NR	Maxillary first molar	276	63
Candeiro et al. (34)	2019	Brazil	NR	Private oral radiology clinic in Fortaleza, Brazil	Cross-sectional and retrospective study	512 (184/328)	44.5 (10–80)	Prexion 3D (Prexion, Inc., San Mateo, USA)	125	NR	Vertucci	Maxillary first molar	700 (237/463)	48.2 (60.2/43.8)
												Maxillary second molar	801 (296/505)	22.7 (30.4/16.8)
Mohara et al. (35)	2019	Brazil	2014–2017	Private radiological clinic, Campinas, São Paulo	Cross-sectional and retrospective study	510 (297/213)	31.4 (18–45)	3D Accuitomo 80 CBCT (J. Morita, Kyoto, Japan)	125	4 × 4 cm/6 × 6 cm (Small)	Zhang and Vertucci	Maxillary first molar	328 (154/174)	64.2 (61/67.2)
												Maxillary second molar	323 (143/180)	33.6 (31.5/46.7)
Martins et al. (12)	2018	World	2017–2018	Campinas	Cross-sectional	127 (53/74)	43.3	i-CAT(i-CAT,Hatfield, England)	200	Large	NR	Maxillary first molar - Brazil	250 (103/147)	82.4 (78.6/85)
				Caracas		250 (105/145)	47.1	CS9000	76	Small	NR	Maxillary first molar - Venezuela	250 (105/145)	48.6 (58.1/40.7)
Gomes Alves et al. (36)	2018	Brazil	2015	São Paulo	Cross-sectional and retrospective study	287 (101/186)	49.4 ± 16.8 (9–93)	Prexion 3D Elite model XP68 (PreXion Inc., San Mateo, California, United State)	150	8.1 × 7.5 cm/5.6 × 5.2 cm (Small)	Vertucci	Maxillary first molar	362 (123/239)	68.2
Betancourt et al. (37)	2017	Chile	2011–2012	Department of Radiology, Faculty of Dentistry, University of La Frontera, Temuco	Cross-sectional	60 (31/29)	26.5 (10–75)	Pax Zenith CBCT (Vatech, Korea, 2011)	120	8 × 6 cm (Medium)	NR	Maxillary first molar	60 (31/29)	68.3 (43.9/56.1)
Calero-Hinostroza et al. (38)	2017	Peru	2016	HANNY X Diagnostic imaging center, Lima	Cross-sectional and retrospective study	120	(18–80)	Point 3D Combi 500 S	NR	12 × 9 cm (Large)	NR	Maxillary second molar	170 (70/100)	40 (44.3/31.3)
Betancourt et al. (39)	2016	Chile	2014–2015	Radiology unit of the University of La Frontera, Temuco	Cross-sectional	NR	(15–75)	Pax Zenith CBCT unit (Vatech, Hwaseong-si, Korea)	120	8 × 6 cm (Medium)	NR	Maxillary first molar	550 (282/268)	69.8 (75.2/64.2)
												Maxillary second molar	550 (278/272)	46.9 (55/38.6)
Falcão et al. (40)	2016	Brazil	2013	Teresina, Piauí state	Cross-sectional and retrospective study	80 (41/39)	45 ± 13	i-CAT Vision	NR	NR	NR	Maxillary first molar	80 (41/39)	56.3 (53.7/56.4)
Estrela et al. (18)	2015	Brazil	2012–2014	Central region of Brazil	Cross-sectional and retrospective study	618 (224/394)	43.4	PreXion 3D Inc. (San Mateo, California, United State)	100	5.6 cm (Small)	NR	Maxillary first molar	100	76
												Maxillary second molar	100	41
Betancourt et al. (17)	2015	Chile	2014	Images from the University of La Frontera service, Temuco	Cross-sectional	NR	(16–75)	Vatech Pax Zenith CBCT machine (Korea, 2011)	120	8 × 6 cm (Medium)	NR	Maxillary second molar	225 (115/110)	48 (63/37)
Abarca et al. (41)	2015	Chile	2010–2013	Begmax Center, Santiago	Cross-sectional and retrospective study	508 (208/300)	33.3 ± 15.6 (8–77)	Gendex CB500 imaging system	200	NR	Vertucci	Maxillary first molar	802 (325/477)	73.4 (76.6/71.3)
												Maxillary second molar	572 (259/313)	42.5 (46.7/39)
Silva et al. (14)	2014	Brazil	2010–2012	University departments in southeastern Brazil (São Paulo and Rio de Janeiro)	Cross-sectional and retrospective study	294 (108/186)	NR	i-CAT System (Imaging Sciences International, Hatfield, Pennsylvania, United State)	200	8 × 8 cm (Medium)	Zhang	Maxillary first molar	314	42.6
												Maxillary second molar	306	34.3
Reis et al. (42)	2013	Brazil	2011	Private clinic in Canoas	Cross-sectional	100 (50/50)	(20–70)	i-CAT 3D imaging device (Imaging Sciences International, Hatfield, Pennsylvania, United State)	200	12 cm (Large)	NR	Maxillary first molar	158	88.5
												Maxillary second molar	185	83.4
Abuabara et al. (43)	2013	Brazil	NR	Curitiba	Cross-sectional	50	(16–65)	i-CAT, Imaging Sciences International, Hatfield, Pennsylvania, United State	120	6 cm (Small)	Vertucci	Maxillary first molar	50	62
Betancourt et al. (44)	2013	Chile	2011–2012	Imaging service of the University of La Frontera, Temuco	Cross-sectional and retrospective study	32 (16/16)	25.3 (10–75)	Pax Zenith, Vatech brand (Korea 2011)	120	8 × 6 cm (Medium)	NR	Maxillary first molar	32 (16/16)	68.8 (68.8/68.8)
Baratto Filho et al. (45)	2009	Brazil	2006–2007	Positivo University (Curitiba) and University of Joinville (Joinville)	Cross-sectional and retrospective study	NR	NR	i-CAT (Imaging Sciences International, Hatfield, Pennsylvania, United State)	200	NR	NR	Maxillary first molar	54	37.1

NR, Not reported.

The sample sizes exhibited considerable variation across the included literature, ranging from 32 to 618 subjects, thereby capturing a broad demographic spectrum. The ages of the evaluated patients ranged widely from 8 to 93 years, although several investigations restricted their inclusion criteria strictly to adult populations (≥ 18 years). Among the selected studies, 21 evaluated the anatomy of permanent maxillary first molars, while 12 assessed permanent maxillary second molars, with several studies reporting data for both (Table 3).

All included investigations utilized CBCT as the sole imaging modality for anatomical assessment; however, the technical parameters varied depending on the clinical center. A diverse array of CBCT devices was employed, with i-CAT models being the most frequently reported. Voxel sizes—a critical determinant for the accurate identification of intricate structures like the MB2 canal—ranged significantly from 76 µm to 500 µm. Furthermore, the field of view (FOV) was highly variable, utilizing small, medium, and large configurations based on the specific acquisition protocols of each study (Table 3).

To evaluate and standardize the reporting of the root canal system's internal morphology, the Vertucci classification was the most widely adopted anatomical framework (explicitly used in 9 studies). A minority of authors opted for the Zhang or Weine criteria. Notably, a considerable proportion of the included studies did not specify the use of any standardized anatomical classification system, focusing exclusively on the binary detection (presence or absence) of the MB2 (Table 3).

### 4.3 Risk of bias analysis of studies

The methodological quality assessment revealed that the vast majority of the included literature is of a high standard. Of the 23 evaluated studies, 21 (91.3%) were classified as having a low risk of bias, demonstrating a high overall methodological quality. Only 2 studies (8.7%) were categorized with a moderate risk of bias due to deficiencies in the reporting of the sampling design and sample size. None of the included studies presented a high risk of bias (Table 4).

**Table 4:** Risk of bias in included studies.

Study	1	2	3	4	5	6	7	8	9	Risk of bias
Blank-Gonçalves et al. (27)	Yes	Yes	Yes	Yes	Yes	Yes	Yes	Yes	N/A	Low
Borges et al. (28)	Yes	Yes	Unclear	Yes	Yes	Yes	Yes	Yes	N/A	Low
Rios et al. (29)	Yes	No	Unclear	Yes	Yes	Yes	Yes	Yes	N/A	Low
Dufey et al. (30)	Yes	Unclear	Yes	Yes	Yes	Yes	Yes	Yes	N/A	Low
Normando et al. (31)	Yes	Yes	Yes	Yes	Yes	Yes	Yes	Yes	N/A	Low
Magalhães et al. (32)	Yes	Yes	Yes	Yes	Yes	Yes	Yes	Yes	N/A	Low
Benazzo et al. (33)	Yes	Unclear	No	Yes	Yes	Yes	Yes	Yes	N/A	Low
Candeiro et al. (34)	Yes	Yes	Unclear	Yes	Yes	Yes	Unclear	Yes	N/A	Low
Mohara et al. (35)	Yes	Yes	Yes	Yes	Yes	Yes	Yes	Yes	N/A	Low
Martins et al. (12)	Yes	Yes	Yes	Yes	Yes	Yes	Yes	Yes	N/A	Low
Gomes Alves et al. (36)	Yes	Yes	Unclear	Yes	Yes	Yes	Yes	Yes	N/A	Low
Betancourt et al. (37)	Yes	Yes	Yes	Yes	Yes	Yes	Yes	Yes	N/A	Low
Calero-Hinostroza et al. (38)	Yes	Unclear	Yes	Yes	Yes	Yes	Yes	Yes	N/A	Low
Betancourt et al. (39)	Yes	Yes	Yes	Yes	Yes	Yes	Yes	Yes	N/A	Low
Falcão et al. (40)	Yes	Yes	No	Yes	Yes	Yes	Unclear	Yes	Yes	Low
Estrela et al. (18)	Yes	Yes	Yes	Yes	Yes	Yes	Yes	Yes	N/A	Low
Betancourt et al. (17)	Yes	Yes	No	Yes	Yes	Yes	Yes	Yes	N/A	Low
Abarca et al. (41)	Yes	Yes	Yes	Yes	Yes	Yes	Yes	Yes	N/A	Low
Silva et al. (14)	Unclear	No	Unclear	Yes	Yes	Yes	Yes	Yes	N/A	Moderate
Reis et al. (42)	Yes	Yes	Yes	Yes	Yes	Yes	Yes	Yes	N/A	Low
Abuabara et al. (43)	No	No	No	Yes	Yes	Yes	Yes	Yes	Yes	Moderate
Betancourt et al. (44)	Yes	Unclear	No	Yes	Yes	Yes	Yes	Yes	N/A	Low
Baratto Filho et al. (45)	Yes	Yes	No	No	Yes	Yes	Yes	Yes	N/A	Low

N/A, Not applicable, 1 = Was the sample frame appropriate to address the target population?, 2 = Were study participants sampled in an appropriate way?, 3 = Was the sample size adequate?, 4 = Were the study subjects and the setting described in detail?, 5 = Was the data analysis conducted with sufficient coverage of the identified sample?, 6 = Were valid methods used for the identification of the condition?, 7 = Was the condition measured in a standard, reliable way for all participants?, 8 = Was there appropriate statistical analysis?, 9 = Was the response rate adequate, and if not, was the low response rate managed appropriately?.

Regarding the domain-specific analysis, the most consistent methodological strengths among the studies were observed in the validity of the identification method (Question 6), the coverage of the data analysis (Question 5), and the appropriateness of the statistical analysis (Question 8), achieving 100% compliance (*n* = 23; “Yes” responses). This is primarily due to the standardized use of CBCT as the gold standard method for anatomical evaluation, alongside the inclusion of all eligible samples in the final results. Furthermore, 22 of the 23 studies (95.7%) described the subjects and the research setting in detail (Question 4), and 21 studies (91.3%) applied standardized and reliable measurements guaranteed through observer calibration (Question 7) (Table 4).

On the other hand, the main methodological deficiencies and sources of bias were related to the sample size calculation (Question 3): only 12 studies (52.2%) adequately justified their sample size, while in 11 studies this aspect was inadequate or unclear. The sampling method (Question 2) was also an area of minor concern, where 7 studies (30.4%) resorted to convenience sampling without proper randomization or presented unclear methods. Finally, because the majority of the studies were retrospective designs based on imaging databases, the criterion regarding the response rate (Question 9) was considered “Not applicable” (N/A) for 21 of the 23 analyzed articles (Table 4).

### 4.4 Synthesis of results

#### 4.4.1 Overall prevalence

The pooled prevalence of the MB2 canal in maxillary molars among the South American population, assessed via CBCT, was estimated at 59% (95% CI: 53%–66%). Extremely high and statistically significant heterogeneity was identified among the studies (I^2^ = 98.1%, *p* < 0.001), strictly justifying the use of a random-effects model (Supplementary Figure S1).

Subgroup analysis by country revealed significant regional variations, highlighting the highest estimates in Argentina at 63% (95% CI: 57%–69%) and Brazil at 62% (95% CI: 53%–72%), followed by Chile at 57% (95% CI: 50%–65%). Conversely, Venezuela and Peru showed lower rates of 49% (95% CI: 43%–55%) and 40% (95% CI: 33%–47%), respectively, each represented by a single study (Supplementary Figure S2). Furthermore, variables such as methodological quality (risk of bias) (Supplementary Figure S3), publication period (Supplementary Figures S4, S5), and technical CBCT parameters—like voxel size (Supplementary Figures S6, S7) and FOV (Supplementary Figure S8)—statistically explained the sample variability. Additionally, a cumulative meta-analysis demonstrated that the overall prevalence rate reached a robust stabilization plateau from 2014 onwards (Supplementary Figure S9).

#### 4.4.2 Tooth-Specific analysis

The isolated evaluation of specific teeth revealed marked anatomical differences. In maxillary first molars, the MB2 canal prevalence solidly rose to 66% (95% CI: 60%–72%), maintaining high heterogeneity (I^2^ = 96.69%, *p* < 0.001) (Supplementary Figure S10). Within this group, studies classified with a low risk of bias reported even higher detection rates, suggesting that greater methodological rigor could be associated with enhanced canal detection capabilities (Supplementary Figure S11). Analysis by country revealed consistent regional variations, with the highest estimates found in Chile at 68% (95% CI: 64%–72%) and Brazil at 67% (95% CI: 58%–77%), followed by Argentina at 63% (95% CI: 57%–69%), while Venezuela showed lower rates at 49% (95% CI: 43%–55%), represented by a single study (Supplementary Figure S12). Unlike the overall analysis, factors such as methodological quality (Supplementary Figure S11), time period (Supplementary Figures S13, S14), voxel size (Supplementary Figures 15, 16), and FOV (Supplementary Figure S17) did not statistically explain the sample variability. A cumulative meta-analysis showed that the prevalence rate reached a stabilization plateau between 2014 and 2015 (Supplementary Figure S18).

In stark contrast, the prevalence in maxillary second molars decreased to 41% (95% CI: 31%–51%), also retaining high heterogeneity (I^2^ = 97.94%, *p* < 0.001) (Supplementary Figure S19). Similar to first molars, low risk of bias studies in this group reported higher detection rates (Supplementary Figure S20). The country-specific analysis showed the highest estimates in Brazil at 43% (95% CI: 26%–60%) and Chile at 39% (95% CI: 26%–62%), while Peru reported a lower rate of 40% (95% CI: 33%–47%) based on a single study (Supplementary Figure S21). Likewise, variability in this group was not significantly explained by methodological quality (Supplementary Figure 20), time period (Supplementary Figures S22, S23), or technical CBCT parameters (Supplementary Figures S24–S26). The cumulative meta-analysis for second molars confirmed prevalence stabilization from 2015 onwards (Supplementary Figure S27).

#### 4.4.3 Sexual dimorphism analysis

The influence of sex on root anatomy varied depending on the tooth. In first molars, sexual dimorphism did not show a statistically significant impact on the presence of the MB2 canal (OR = 1.08, 95% CI: 0.95–1.22, *p* = 0.24) (Supplementary Figure S28). However, the analysis focused on second molars revealed a significant disparity, determining that male patients are 35% more likely to present this canal compared to females (OR = 1.35, 95% CI: 1.09–1.67, *p* = 0.01) (Supplementary Figure S29).

#### 4.4.4 Sensitivity analysis and publication bias

The statistical model proved to be highly robust. Leave-one-out sensitivity analyses confirmed that the overall and first molar estimates remained highly stable against the iterative exclusion of individual studies, without altering the significance of the main findings (Supplementary Figure S30, S31). Nevertheless, in second molars, the pooled estimate was found to be strongly influenced by an outlier study (42) that reported an 83% prevalence. The analysis demonstrated that upon excluding this study, the true prevalence in second molars would converge towards 37% (Supplementary Figure S32).

Finally, the symmetry of the funnel plots, mathematically corroborated by Egger's and Harbord's tests (all *p*-values > 0.05) (Supplementary Figures S33–S38), conclusively ruled out the presence of publication bias, guaranteeing that current South American literature objectively reports the full spectrum of anatomical findings.

### 4.5 GRADE analysis

The certainty of the body of evidence for the meta-analytical findings was assessed using the GRADE approach. The detailed evaluation by outcomes yielded results ranging from moderate to high certainty. (Table 5).

**Table 5:** GRADE analysis of included studies.

Certainty assessment										Certainty
№ of studies	Study design	Risk of bias	Inconsistency	Indirectness	Imprecision	Publication bias	Magnitude of effect	Dose-response gradient	Plausible confounding	
Prevalence of the MB2 canal in upper molars										
23	Observational	Not serious	Not serious	Not serious	Not serious	Not serious	Not applicable	Not applicable	Not applicable	⊕⊕⊕⊕ High
Prevalence of the MB2 canal in upper first molars										
21	Observational	Not serious	Not serious	Not serious	Not serious	Not serious	Strong	Not applicable	Not applicable	⊕⊕⊕⊕ High
Prevalence of the MB2 canal in upper second molars										
11	Observational	Not serious	Serious	Not serious	Serious	Not serious	Strong	Not applicable	Not applicable	⊕⊕⊕⃝ Moderate

#### 4.5.1 Overall prevalence in maxillary molars

The certainty of the evidence for the overall prevalence of the MB2 canal was rated as High. This rating is supported by the fact that the vast majority of the included studies presented a low risk of bias, reporting a prevalence closely aligned with the overall estimate (59%). Although high statistical heterogeneity was recorded (I^2^ = 98.1%), the inconsistency domain was not penalized because this variability is inherent and expected in anatomical prevalence studies, and it was adequately controlled using a random-effects meta-analysis model. Furthermore, clinical applicability was direct as the *in vivo* reference standard (CBCT) was used. The precision of the results was guaranteed through narrow confidence intervals and a sensitivity analysis (leave-one-out) that demonstrated high stability in the estimate (fluctuating minimally between 58% and 61%). Finally, Egger's test (*p* = 0.745) corroborated the mathematical symmetry of the funnel plot, ruling out publication bias (Table 5).

#### 4.5.2 Prevalence in maxillary first molars

When isolating the analysis to maxillary first molars, the quality of evidence remained High. For this tooth, investigations with greater methodological rigor solidly supported the findings, detecting higher prevalences that reached 69%. The uncertainty generated by heterogeneity (I^2^ = 96.69%) was mitigated by observing in the cumulative meta-regression that the prevalence reached a consensus and solid stabilization (65% - 67%) since 2014. The evidence proved to be mathematically precise and highly robust; the iterative exclusion of individual studies barely caused the mean to fluctuate (66% to 68%). No reporting bias was identified (Egger *p* = 0.1257), and a strong magnitude of clinical effect was highlighted, establishing that the presence of this canal constitutes an overwhelming anatomical constant in this root (Table 5).

#### 4.5.3 Prevalence and sexual dimorphism in maxillary second molars

In stark contrast, the overall certainty of evidence for maxillary second molars was downgraded to Moderate. This downgrade was due to two main factors: first, serious inconsistency was identified, caused by the severe exacerbation of anatomical variability due to an extreme outlier study, which reported an unusual 83% detection rate. Second, serious imprecision confined to the overall absolute prevalence (41%) was determined; the sensitivity analysis revealed notable mathematical fragility, since, upon removing this outlier study, the pooled estimate dropped sharply to 37%. Despite this, no asymmetry or publication bias was evident (Egger p = 0.3922) (Table 5).

It is important to highlight that the subgroup analysis focused on sexual dimorphism for this tooth managed to rescue a strong magnitude of effect and lower statistical heterogeneity (I^2^ = 60.57%). Thus, with solid evidence and no reporting biases (Harbord's test *p* = 0.917), it was determined that males have a 35% higher anatomical probability of possessing an MB2 canal compared to females (Table 5).

## 5 Discussion

The main objective of this systematic review and meta-analysis was to determine the prevalence of the MB2 canal in permanent maxillary molars within the South American population. The findings categorically confirm that a substantial proportion presents this variation, constituting an anatomical constant that manifests in 59% of the evaluated population and reaches its maximum phenotypic expression in the first molars. Beyond statistical precision, this result has profound practical clinical relevance: the inability to properly locate, diagnose, and treat the MB2 canal is historically considered one of the main causes of endodontic failure, as it promotes microbial persistence and the development of apical periodontitis (13, 15). Consequently, the contemporary endodontist must assume the existence of the complex MB2 system as the baseline anatomical norm and abandon blind tactile explorations, definitively transitioning toward the protocolized use of optical magnification and three-dimensional imaging.

When contrasting our data with the global literature, the results robustly coincide with international trends, although they evidence particularities that point to the geographical area as a determining evolutionary factor. The review by Martins et al. (15) calculated a pooled overall prevalence of 69.6% for first molars and 39.0% for second molars, Supplementary Figures virtually identical to the 66% and 41% rates estimated in our South American sample. Likewise, studies in Asian and Iranian populations (1, 4, 13) strengthen the clear pattern that the MB2 is housed much more frequently in the first molar. However, we observed a marked and singular intra-regional variability: The Southern Cone countries (Argentina and Brazil) boast the highest epidemiological estimates, while Andean or northern populations of the region, such as Peru and Venezuela, exhibited considerably lower rates, strongly suggesting that global population results should not be extrapolated without caution.

It is crucial to consider that the severe statistical heterogeneity detected (I^2^ = 98.1%) stems not only from demographic differences but also from the diagnostic methodologies applied. The substantial heterogeneity observed across studies (I^2^ = 98.1%) is not unexpected in multicenter anatomical prevalence studies. Unlike therapeutic interventions, anatomical prevalence is strongly influenced by intrinsic population characteristics, including genetic background, ethnic admixture, craniofacial morphology, and environmental factors, as well as methodological variability among studies. Therefore, a high degree of between-study variability reflects genuine biological and methodological differences rather than inconsistency in the underlying evidence. Consequently, the random-effects model was considered the most appropriate analytical approach to account for this expected variability and provide more generalizable pooled estimates. Our analyses demonstrated that the technical parameters of the CBCT, such as voxel size and FOV, do not statistically explain the entire wide variance, despite external studies warning that lower spatial resolution masks the fragile anatomy of the MB2 (46), while others suggest that a voxel ≤ 200 µm is sufficient (15). Nevertheless, the evaluation method strongly modulates detection rates; although CBCT is the non-destructive gold standard, it's *in vivo* clinical combination with the dental operating microscope and ultrasonic instrumentation has been shown to raise detection rates to levels of 71.9% to 78% (1, 4). Additionally, it was proven that greater methodological rigor has a direct impact, given that South American studies categorized with a low risk of bias reported superior anatomical detection capabilities.

From a theoretical and biological framework, geographical differences and the drastic reduction in prevalence from the first to the second molar can be justified through anthropological, evolutionary, and genetic dynamics. Evidence suggests that populations historically subjected to strong mechanical demands maintained larger teeth and, consequently, greater canal complexity, unlike Eurasian populations that experienced root simplification driven by early dietary modifications (4, 15). Perhaps one of the most eloquent biological findings lies in the evident sexual dimorphism for the second molar, where we verified that men have a 35% higher anatomical probability of presenting this canal. This disparity resonates with the systematic literature and is attributed to the differential effect of the X and Y chromosomes on dentin morphogenesis, resulting in crowns and roots of larger buccolingual dimensions in males, which presumably delays total calcification and favors the spatial and radiographic persistence of the MB2 (13, 15).

Despite the high methodological level of this review, it is necessary to outline its limitations transparently. Primarily, substantial statistical heterogeneity (I^2^ > 90%) was observed, a common finding in anatomical prevalence meta-analyses that include populations with different genetic backgrounds, geographic origins, and CBCT acquisition protocols (4, 13). Although this variability was explored through subgroup analyses, meta-regression, and sensitivity analyses, a considerable proportion remained unexplained, suggesting that additional biological and methodological factors may contribute to the observed differences. Particularly in second molars, we identified that the global pooling (41%) was susceptible to a distortion bias caused by an atypical study that spiked detection to an implausible 83%; our iterative sensitivity analyses demonstrated that, upon excluding it, the true prevalence would converge around 37%, which justified downgrading the certainty of the evidence to a “Moderate” level (GRADE). Additionally, the Achilles heel of regional primary literature remains descriptive statistics: there is a recurrent lack of formal sample size calculation (insufficient in 47.8%) and the inability to thoroughly stratify data by age groups, a key variable due to gradual obliteration by secondary dentin apposition (1, 4). Fortunately, our primary strength lies in having consolidated specific information for South America, backed by studies of high methodological quality (91.3%) and symmetrical funnel plots that eradicated any suspicion of publication bias.

The practical implications of this work transcend the morphological debate to settle into real-world prognostic success as an invaluable prophylactic tool. Recent endodontic research in the South American region reports with concern that nearly 70% of MB2 canals that go unnoticed clinically and radiographically end up harboring post-treatment chronic apical periodontitis (47). In this regard, it is urgent to standardize protocols that incorporate the routine use of CBCT and optical magnification when intervening in the anatomy of the upper maxilla, especially in male patients (1, 13). Likewise, future researchers are urged to unify criteria by adopting universally validated classification systems, such as Vertucci's, to bridge the profound bibliographical gap in Andean subpopulations, and to systematically consider detailed reports on age and sex. In conclusion, we state with high certainty that the MB2 canal is a structurally frequent and clinically significant variation (59%) in the South American population, and its predictive approach through advanced technologies is determinant for transforming and increasing success rates in South American endodontic practice.

## 6 Conclusions

In conclusion, the present systematic review and meta-analysis demonstrates, with a high level of certainty, that the MB2 is a highly prevalent anatomical configuration in the maxillary molars of the South American population, present in approximately six out of ten teeth evaluated using CBCT. Specifically, in maxillary first molars, the evidence (also of high certainty) confirms that the presence of the MB2 constitutes the anatomical norm rather than the exception, reaching a prevalence rate of 66%, a Supplementary Figure that is even more consistent in investigations with a low risk of methodological bias.

Conversely, the prevalence of the MB2 canal in maxillary second molars decreases significantly, being estimated at 41%. This estimate carries a moderate level of certainty, justified by a higher statistical sensitivity to outliers in the literature. However, an important demographic association was identified in this specific tooth: a marked sexual dimorphism exists, wherein male patients have a 35% higher probability of possessing the canal compared to female patients.

Finally, the results demonstrate that the South American literature regarding this anatomical parameter is chronologically mature, having stabilized since the middle of the last decade. Furthermore, it was statistically confirmed that technical variations such as FOV or voxel resolution (ranging from 76 to 500 µm) do not significantly alter the epidemiological detection rates of the canal. From a clinical perspective, these findings dictate that endodontic professionals must maintain a systematically high index of suspicion when approaching maxillary molars, relying on CBCT as a fully validated diagnostic tool to predict this high anatomical complexity.

## Data Availability

The original contributions presented in the study are included in the article/Supplementary Material, further inquiries can be directed to the corresponding author.
