# Supplementary material for: Prevalence of the second mesiobuccal canal of maxillary molars in the South American population: a systematic review and meta-analysis of CBCT studies
Source: Front Dent Med. 2026 Aug 31;7:1928424. doi: 10.3389/fdmed.2026.1928424 (PMC13572276; doi:10.3389/fdmed.2026.1928424)
Supplement: Supplementary file 1 [file Datasheet1.docx]

Supplementary Figures (1-38) - Prevalence of the second mesiobuccal canal of maxillary molars in the South American population: A systematic review and meta-analysis of CBCT studies

Supplementary Figure S1:

Supplementary Figure S2:

Supplementary Figure S3:

Supplementary Figure S4:

Supplementary Figure S5:

Supplementary Figure S6:

Supplementary Figure S7:

Supplementary Figure S8:

Supplementary Figure S9:

Supplementary Figure S10:

Supplementary Figure S11:

Supplementary Figure S12:

Supplementary Figure S13:

Supplementary Figure S14:

Supplementary Figure S15:

Supplementary Figure S16:

Supplementary Figure S17:

Supplementary Figure S18:

Supplementary Figure S19:

Supplementary Figure S20:

Supplementary Figure S21:

Supplementary Figure S22:

Supplementary Figure S23:

Supplementary Figure S24:

Supplementary Figure S25:

Supplementary Figure S26:

Supplementary Figure S27:

Supplementary Figure S28:

Supplementary Figure S29:

Supplementary Figure S30

Supplementary Figure S31:

Supplementary Figure S32:

Supplementary Figure S33:

Egger's test =0.745

Supplementary Figure S34:

Egger's test =0.1257

Supplementary Figure S35:

Egger's test =0.3922

Supplementary Figure S36:

Harbord's test =0.8258

Supplementary Figure S37:

Harbord's test =0.6458

Supplementary Figure S38:

Harbord's test =0.9178
